# Supplementary material for: Analysis of PPARγ Signaling Activity in Psoriasis
Source: Int J Mol Sci. 2021 Aug 10;22(16):8603. doi: 10.3390/ijms22168603 (PMC8395241; doi:10.3390/ijms22168603)
Supplement: Supplementary file 1 [file ijms-22-08603-s001.zip › Supplemental materials_Analysis of PPARg signaling activity in psoriasis/Pathway models/Models images and html files/Model of PPARG signaling in psoriasis/69971.html]

IL33


# Protein IL33

|  |  |
| --- | --- |
| URN | urn:agi-llid:90865 |
| Total Entities | 0 |
| Connectivity | 3788 |
| Name | IL33 |
| Description | interleukin 33 |
| Notes | The protein encoded by this gene is a cytokine that binds to the IL1RL1/ST2 receptor. The encoded protein is involved in the maturation of Th2 cells and the activation of mast cells, basophils, eosinophils and natural killer cells. Several transcript variants encoding different isoforms have been found for this gene. [provided by RefSeq, Sep 2015] |
| Primary Cell Localization | Extracellular |
| Class | Ligand |
| ObjectType | Protein |

---

|  |  |
| --- | --- |
| Pathway | NOD-like Receptors |
|  | Toll-like Receptor Independent Sterile Inflammation |
|  | Basophil Activation |
|  | Eosinophil Survival by Cytokine Signaling |
|  | Eosinophil Activation |
|  | Mast-Cell Activation without Degranulation |
|  | Mast-Cell Activation without Degranulation through IL33/IL1RL1 Signaling |
|  | Proteins Involved in Myocarditis |
|  | Mast-Cells Activation in Atopic Dermatitis |
|  | Acute Phase in Atopic Dermatitis |
|  | Proteins with Altered Expression in Atopic Dermatitis |
|  | Atopic Dermatitis |
|  | Apoptotic Keratinocytes Clearance Recession in Systemic Lupus Erythematosus |
|  | Dendritic Cell Activation in Systemic Lupus Erythematosis |
|  | Plasmacytoid Dendritic Cell in Diabetes Mellitus Type 1 |
|  | Conventional Dendritic Cell Role in Diabetes Mellitus Type 1 |
|  | Macrophage Dysfunction |
|  | Epithelial Cell in the Innate Immune Response in Ulcerative Colitis |
|  | Proteins Involved in Ulcerative Colitis |
|  | Eosinophil Survival in Asthma |
|  | IL33 Signaling Related Eosinophilia in Asthma |
|  | IL17F Signaling in Bronchial Epithelial Cell in Asthma |
|  | Basophil Activation in Asthma |
|  | Antigen-Presenting Cell Role in Asthma |
|  | Dendritic Cell Dysfunction in Psoriatic Arthritis |
|  | Proteins Involved in Rheumatoid Arthritis |
|  | PAPA Syndrome |
|  | Mevalonate Kinase Deficiency |
|  | IL36 Receptor Antagonist (IL1F5) Deficiency (DITRA) |
|  | Cryopyrin (NLPR3) Associated Periodic Syndromes |
|  | Familial Mediterranean Fever |
|  | Proteins Involved in Osteoporosis |
|  | Proteins Involved in Spontaneous Abortion |
|  | Proteins Involved in Age-Related Macular Degeneration |
|  | Immune System Role in Otitis Media |
|  | Proteins with Altered Expression in Asthma |
|  | Elevated Receptors -> Expression Targets in Mouth |
|  | Elevated Receptors -> Expression Targets in Lymphoid System and Blood |
|  | Elevated Receptors -> Expression Targets in Lung |
|  | e |
|  | pathway 4 |
|  | pprarg neg, uknown targets, ps-positive |
|  | Model of PPARG signaling in psoriasis |
|  | dsRNA induces innate immunity and INFs in alveolar cells |
|  | Dendritic Cell Activation in COVID19 |
|  | 2\_Acute phase initiation in atopic dermatitis |
|  | 1\_Th2 cell response in asthma |
|  | 3\_2\_Regulation of airway surface liquid (ASL) thickness |
|  | 2\_2\_Eosinophilia and neutrophilia in asthma: eosinophil apoptosis |
|  | 4\_2\_Airway smooth muscle cells proliferation and airway tissue remodeling |
|  | 1\_Defects in response to pathogens in the gut promotes inflammation in Crohn's disease |
|  | 4\_2\_Genes with mutations currently associated with ulcerative colitis |
|  | 2\_Intestine epithelial cells drive immunological responses in ulcerative colitis |
|  | 4\_1\_Polymorphisms associated with inflammatory bowel diseases |
|  | 1\_Antigen-presenting cells promote islet dysfunction and immune system activation in T1D |
|  | Figure 4\_Diabetes mellitus Type 1: conventional dendritic cell |

---

|  |  |
| --- | --- |
| Group | Genes with Mutations in GWA Studies of Asthma |

---

|  |  |
| --- | --- |
| MedScan ID | 90865 |

---

|  |  |
| --- | --- |
| LocusLink ID | 90865 |
|  | 77125 |
|  | 361749 |

---

|  |  |
| --- | --- |
| Alias | chromosome 9 open reading frame 26 (nf-hev) |
|  | nuclear factor for high endothelial venules |
|  | 9230117N10Rik |
|  | IL33 |
|  | 9230117N10 |
|  | DVS27 |
|  | RGD1311155 |
|  | chromosome 9 open reading frame 26 |
|  | OTTHUMP00000021041 |
|  | interleukin-1 family member 11 |
|  | NF-HEV |
|  | IL1f11 |
|  | RP11-575c20.2 |
|  | OTTMUSP00000035769 |
|  | DKFZp586H0523 |
|  | interleukin IL1f11 |
|  | NFEHEV |
|  | C9orf26 |
|  | interleukin IL33 |
|  | nuclear factor from high endothelial venules |
|  | interleukin 33 |
|  | DVS27-related protein |
|  | interleukin-33 |
|  | Il-33 |
|  | NFHEV |
|  | IL-1F11 |

---

|  |  |
| --- | --- |
| GO ID | 0005125 |
|  | 0051607 |
|  | 0097191 |
|  | 0038172 |
|  | 0002282 |
|  | 0061518 |
|  | 0002826 |
|  | 0051025 |
|  | 0032689 |
|  | 0002686 |
|  | 0090197 |
|  | 0051024 |
|  | 0050729 |
|  | 0032736 |
|  | 0032753 |
|  | 0032754 |
|  | 0032755 |
|  | 0043032 |
|  | 0150078 |
|  | 0032436 |
|  | 0045944 |
|  | 0002830 |
|  | 0016579 |
|  | 0005694 |
|  | 0005576 |
|  | 0005615 |
|  | 0005654 |
|  | 0030133 |
|  | 0002281 |
|  | 0010628 |
|  | 0031410 |
|  | 0005634 |
|  | 0010469 |
|  | 0042092 |
|  | 0006351 |
|  | 0005515 |
|  | 0001819 |

---

|  |  |
| --- | --- |
| KEGG ID | hsa:90865 |
|  | mmu:77125 |
|  | rno:361749 |

---

|  |  |
| --- | --- |
| Organism | Homo sapiens {Organism urn:agi-taxid:9606} |
|  | Mus musculus {Organism urn:agi-taxid:10090} |
|  | Rattus norvegicus {Organism urn:agi-taxid:10116} |
|  | Homo sapiens |
|  | Mus musculus |
|  | Rattus norvegicus |

---

|  |  |
| --- | --- |
| Mouse chromosome position | 19 |

---

|  |  |
| --- | --- |
| OMIM ID | 608678 |

---

|  |  |
| --- | --- |
| Rat chromosome position | 1q52 |

---

|  |  |
| --- | --- |
| Hugo ID | 16028 |
|  | HGNC:16028 |

---

|  |  |
| --- | --- |
| Human chromosome position | 9p24.1 |

---

|  |  |
| --- | --- |
| Swiss-Prot Accession | O95760 |
|  | O95760.1 |
|  | Q8BVZ5 |
|  | Q8BVZ5.1 |
|  | Q66H70 |
|  | Q66H70.1 |
|  | B2R8L1 |
|  | B4DJ35 |
|  | B4E1Q9 |
|  | D3DRI5 |
|  | E7EAX4 |
|  | Q2YEJ5 |
|  | Q2YEJ4 |
|  | Q3TQN0 |
|  | Q99L46 |

---

|  |  |
| --- | --- |
| GenBank ID | NC\_000009 |
|  | XM\_017015285 |
|  | XP\_016870774 |
|  | NM\_001314044 |
|  | NP\_001300973 |
|  | NM\_001314047 |
|  | NP\_001300976 |
|  | NM\_001199641 |
|  | NP\_001186570 |
|  | NM\_033439 |
|  | NP\_254274 |
|  | NM\_001314046 |
|  | NP\_001300975 |
|  | NM\_001199640 |
|  | NP\_001186569 |
|  | NM\_001314048 |
|  | NP\_001300977 |
|  | NM\_001314045 |
|  | NP\_001300974 |
|  | NM\_001353802 |
|  | NP\_001340731 |
|  | NG\_047209 |
|  | AL353741 |
|  | CH471071 |
|  | EAW58748 |
|  | EAW58749 |
|  | EAW58750 |
|  | EAW58751 |
|  | AB024518 |
|  | BAA75892 |
|  | AI610794 |
|  | AK295908 |
|  | BAG58697 |
|  | AK303943 |
|  | BAG64871 |
|  | AK313414 |
|  | BAG36208 |
|  | AL080211 |
|  | AY905581 |
|  | AAX86998 |
|  | BC047085 |
|  | AAH47085 |
|  | BQ878541 |
|  | CR407619 |
|  | CAG28547 |
|  | DA439244 |
|  | DA713547 |
|  | DA719342 |
|  | DB199599 |
|  | DB266668 |
|  | DB477860 |
|  | HQ641439 |
|  | ADR77828 |
|  | KX463463 |
|  | AOZ26494 |
|  | KX463464 |
|  | AOZ26495 |
|  | O95760 |
|  | NC\_000085 |
|  | NM\_133775 |
|  | NP\_598536 |
|  | XM\_011247399 |
|  | XP\_011245701 |
|  | NM\_001164724 |
|  | NP\_001158196 |
|  | XM\_006527463 |
|  | XP\_006527526 |
|  | NM\_001360725 |
|  | AC111048 |
|  | AC157785 |
|  | CH466534 |
|  | EDL41694 |
|  | AK020353 |
|  | AK030502 |
|  | AK075849 |
|  | BAC36003 |
|  | AK163464 |
|  | BAE37352 |
|  | AY905582 |
|  | AAX86999 |
|  | BC003847 |
|  | AAH03847 |
|  | CJ191135 |
|  | HQ176370 |
|  | ADW82003 |
|  | Q8BVZ5 |
|  | NC\_005100 |
|  | XM\_008760344 |
|  | XP\_008758566 |
|  | NM\_001014166 |
|  | NP\_001014188 |
|  | AC\_000069 |
|  | AABR07006687 |
|  | AAHX01000037 |
|  | AAHX01000038 |
|  | CH473953 |
|  | EDM13106 |
|  | EDM13107 |
|  | BC081993 |
|  | AAH81993 |
|  | FQ212643 |
|  | Q66H70 |
|  | ABBA01065398 |
|  | AABR06010499 |
|  | AMYH02019091 |
|  | XP\_005251683 |
|  | XP\_005251684 |
|  | AC\_000041 |
|  | XM\_011518060 |
|  | XM\_011518061 |
|  | XP\_011516362 |
|  | XP\_011516363 |
|  | AC\_000141 |
|  | XP\_011516361 |
|  | NC\_018920 |
|  | XM\_005251626 |
|  | AABR06010500 |
|  | XM\_005251627 |
|  | AAHY01142995 |
|  | AAHY01142996 |
|  | AAHY01142994 |
|  | CAI16003 |
|  | XM\_011518059 |
|  | NT\_008413 |
|  | NW\_001839149 |
|  | NW\_924062 |
|  | AC\_000052 |
|  | NT\_039687 |
|  | NW\_001030643 |
|  | NW\_047565 |
|  | NW\_001084774 |

---

|  |  |
| --- | --- |
| Swiss-Prot ID | IL33\_HUMAN |
|  | IL33\_MOUSE |
|  | IL33\_RAT |
|  | B4DJ35\_HUMAN |
|  | B4E1Q9\_HUMAN |

---

|  |  |
| --- | --- |
| Cell Localization | Nucleus |
|  | Chromosome |
|  | Cytoplasmic vesicle |
|  | Secretory vesicle |
|  | Secreted |
|  | Extracellular |
|  | Cytoplasmic vesicle, secretory vesicle |

---

|  |  |
| --- | --- |
| Ensembl ID | ENSG00000137033 |
|  | ENSP00000414238.2 |
|  | ENST00000456383.3 |
|  | ENSP00000394039.2 |
|  | ENST00000417746.6 |
|  | ENSP00000370842.3 |
|  | ENST00000381434.7 |
|  | ENSP00000478858.1 |
|  | ENST00000611532.4 |
|  | ENSMUSG00000024810 |
|  | ENSMUSP00000025724.8 |
|  | ENSMUST00000025724.8 |
|  | ENSMUSP00000113829.2 |
|  | ENSMUST00000120388.8 |
|  | ENSRNOG00000016456 |
|  | ENSRNOP00000022056.6 |
|  | ENSRNOT00000022056.7 |
|  | ENST00000381434 |
|  | ENST00000456383 |
|  | ENSMUST00000025724 |
|  | ENSMUSP00000025724 |
|  | ENSRNOP00000022056 |
|  | ENSMUSP00000113829 |
|  | ENSP00000414238 |
|  | ENSRNOT00000022056 |
|  | ENSP00000394039 |
|  | ENST00000417746 |
|  | ENSP00000370842 |
|  | ENSMUST00000120388 |

---

|  |  |
| --- | --- |
| MGI ID | MGI:1924375 |
|  | 1924375 |

---

|  |  |
| --- | --- |
| RGD ID | 1311155 |

---

|  |  |
| --- | --- |
| Unigene ID | Mm.182359 |
|  | Hs.731660 |
|  | Rn.106849 |
|  | Hs.348390 |

---

|  |  |
| --- | --- |
| Homologene ID | 14126 |

---

|  |  |
| --- | --- |
| Shape | Rhomb |

---

|  |  |
| --- | --- |
| IPI ID | IPI00471802 |
|  | IPI00908682 |
|  | IPI00116304 |
|  | IPI00027628 |
|  | IPI00909430 |
|  | IPI00882194 |

---
